# Safety notices and registry outlier data measure different aspects of safety and performance of total knee implants: a comparative study of safety notices and register outliers

**DOI:** 10.2340/17453674.2024.42361

**Published:** 2024-11-25

**Authors:** Lotje A HOOGERVORST, Yijun REN, Tom MELVIN, Ashley A STRATTON-POWELL, Anne LÜBBEKE, Robert E GEERTSMA, Alan G FRASER, Rob G H H NELISSEN, Enrico G CAIANI, Perla J MARANG-VAN DE MHEEN

**Affiliations:** 1 Department of Orthopaedics, Leiden University Medical Center, Leiden, the Netherlands; 2 Department of Electronics, Information and Biomedical Engineering, Politecnico di Milano, Milan, Italy; 3 School of Medicine, Trinity College Dublin, Dublin, Ireland; 4 Division of Orthopaedic Surgery and Traumatology, Geneva University Hospitals and University of Geneva, Geneva, Switzerland; 5 Nuffield Department of Orthopaedics, Rheumatology and Musculoskeletal Sciences, University of Oxford, Oxford, UK; 6 Centre for Health Protection, National Institute for Public Health and the Environment, Bilthoven, the Netherlands; 7 Department of Cardiology, University Hospital of Wales, Heath Park Way, Cardiff, UK; 8 Istituto Auxologico Italiano IRCCS, Milan, Italy; 9 Safety & Security Science and Centre for Safety in Healthcare, Delft University of Technology, Delft, the Netherlands

## Abstract

**Background and purpose:**

Safety notices for medical devices such as total knee arthroplasty (TKA) implants may indicate problems in their design or performance that require corrective action to prevent patient harm. Safety notices are often published on national Ministries of Health or regulatory agencies websites. It is unknown whether problems triggering safety notices identify the same implants as those identified by registries as “outlier.” We aimed to assess the extent to which safety notices and outlier identification in registries signal the same or different TKA implants.

**Methods:**

The CORE-MD tool, an automated web scraper tool, was used to collect safety notices related to TKA implants on 13 national Ministries of Health websites and regulatory agencies. Safety notices were defined according to the Medical Device Regulation (MDR) as “a communication sent by a manufacturer to users or customers in relation to a field safety corrective action.” Identified TKA outliers, defined as having a significantly higher revision risk than other comparable TKA implants, were extracted from registry reports.

**Results:**

787 safety notices for 38 TKA implants and 35 TKA outliers were identified, together identifying 47 unique TKA implants. 26 (55%) TKA implants had safety notices and were also outliers, 12 (26%) TKA implants had only safety notices, and 9 (19%) were outliers only. TKA implants with safety notices only had similar types of problems to TKA outliers with safety notices, with “Manufacturing/Packaging/Shipping” problems being most frequent (44%). Cumulative revision risks (1/5/10 years) were lower for TKA implants with safety notices only than for TKA outliers with safety notices.

**Conclusion:**

55% of the TKA with a safety notice were identified as outliers in the registry, whereas around 25% of TKA outliers are not the subject of publicly released safety notices, with safety notices pointing to TKA implants not identified by registries as potentially having a higher risk of failure. This suggests that safety notices and registry outlier data measure different aspects of safety and performance.

Medical devices are subject to post-market surveillance (PMS) where manufacturers have to collect and review data on experience with their devices [1]. Once collected, these data must be analyzed by the manufacturer to evaluate if any corrective or preventive actions are needed. If action is required to prevent patient harm, a safety notice must be issued [2]. Safety notices can be published on the websites of manufacturers, Ministries of Health, and regulatory agencies. Safety notices may include a recall, amended instructions for use, adverse events, or additional information concerning the device. From a safety and performance perspective, total knee arthroplasty (TKA) implants are of interest, as together with total hip implants they are the most used arthroplasty implants.

Safety notices are relevant for clinicians and hospitals as they may guide implant selection. Safety notices can be issued for a wide variety of implant-related issues (e.g., packaging and labeling), which are not always associated with the safety or performance of a TKA implant. On the other hand, several arthroplasty registries have procedures in place to identify TKA implants with outlier performance (i.e., a significantly higher revision risk than other comparable implants) [3], defined solely based on revision risk [4,5]. Safety notices, however, may also refer to signals based on other outcomes (e.g., poor patient satisfaction scores), meaning that safety notices and outlier identification may reflect different aspects of patient safety. Furthermore, safety notices may be issued based on other data sources such as peer-reviewed publications. Hence, it is unknown whether problems triggering safety notices identify the same TKA implants as those identified by registries as outliers.

We aimed to assess the extent to which safety notices and outliers identified by registries signal the same or different TKA implants, and to explore possible reasons for any discrepancies.

## Methods

### Study design

This study focused on the agreement between 2 real-world data sources that are intended to signal problems related to medical devices, and more specifically to assess whether TKA implants for which safety notices were published on the websites of Ministries of Health and regulatory agencies were the same as the TKA implants identified and publicly reported by registries as outliers. Only TKA implants currently used on the market were included. The study was conducted according the to the STARD guidelines [6].

### Data collection of safety notices reporting TKA implants

The Coordinating Research and Evidence for Medical Devices (CORE-MD) PMS tool [7], an automated web scraper tool, was used to identify TKA implants with safety notices on the websites of Ministries of Health and regulatory agencies. 13 countries were included in the CORE-MD tool and were therefore assessed in the current study: Australia, Czechia, Denmark, France, Germany, Greece, Ireland, Italy, Portugal, Spain, Sweden, the United States of America (USA), and the Netherlands. Note that all historical and publicly available safety notices were retrieved for each country with their respective last update (Supplementary Table 1).

Details of the applied methodology in the CORE-MD PMS tool have been published [7]. Briefly, the tool screens the website of each Ministry of Health and regulatory agency to collect all safety information, including safety notices, alerts, and recalls. We refer to safety notices to indicate the collective safety information found on these websites.

To include only safety notices for TKA implants currently on the market, a list of all TKA implants from the latest annual reports from the following national and regional registries was constructed: American Joint Replacement Registry (AJRR) [8], Australian Orthopaedic Association National Joint Replacement Registry (AOANJRR) [9], Dutch Arthroplasty Register (LROI) [10], Emilia-Romagna Register (RIPO) [11], German Arthroplasty Registry (EPRD) [12], Swiss National Hip & Knee Joint Registry (SIRIS) [13], and the National Joint Registry for England, Wales, Northern Ireland, the Isle of Man and Guernsey (NJR) [14], and up-to-date registry-website data from the Finnish Arthroplasty Register (FAR) [15]. Note that some countries included in the CORE-MD PMS tool to identify safety notices are not used to construct the list of TKA implants currently on the market as they lack a(n) (active) regional or national arthroplasty registry capturing data on TKA implants [3]. We assumed that safety notices would identify problems that relate to the implant itself rather than reflecting, e.g., limited experience by surgeons or patient case-mix, and thereby that the problems highlighted in these countries would reflect problems elsewhere.

The brand name of each TKA implant on this list was used as input for the CORE-MD PMS tool, to extract all associated safety notices for further analysis. Based on the extended safety notice text, the described adverse event was linked to an International Medical Device Regulators Forum (IMDRF) medical device problem code [16]. These IMDRF codes have a hierarchical alphanumerical coding structure, including a letter (i.e., referring to the Annex A in our case) followed by numerical codes at different levels of detail [16,17]. Level 1 terms are represented by the first 2 digits, referring to 27 different medical device problems (Table 1). Level 2 and 3 terms are described by the digits 3 to 4 and 5 to 6 respectively, representing a more detailed description of the problem under 1 of the overarching 27 groups. In this study only the Level 1 terms were used, as they are already detailed enough to distinguish different device problems. All safety notices related to TKA implants were independently classified to an IMDRF code by 2 researchers (LH and YR); possible discrepancies in coding were resolved by discussion. To determine interobserver variability, the Cohen’s kappa (κ) was calculated. Kappa values were categorized into 6 levels: (i) κ ≤ 0 (no agreement); (ii) κ = 0.01–0.20 (none to slight); (iii) κ = 0.21–0.40 (fair); (iv) κ = 0.41–0.60 (moderate); (v) κ = 0.61–0.80 (substantial), and (vi) κ = 0.81–1.00 (almost perfect) (18). Analysis was performed using Python (version 3.11.5; https://www.python.org/downloads/release/python-3115/).

**Table 1:** The 27 International Medical Device Regulators Forum (IMDRF) medical device problem codes and relevant description (15)

IMDRF code	IMDRF description of medical device problem
A01 – Patient Device Interaction Problem	Problem related to the interaction between the patient and the device.
A02 – Manufacturing, Packaging or Shipping Problem	Problem associated with any deviations from the documented specifications of the device that relate to nonconformity during manufacture to the design of an item or to specified manufacturing, packaging, or shipping processes (out of box problem).
A03 – Chemical Problem	Problem associated with any deviations from the documented specifications of the device that relate to any chemical characterization, i.e., element, compound, or mixture.
A04 – Material Integrity Problem	Problem associated with any deviations from the documented specifications of the device that relate to the limited durability of all material used to construct device.
A05 – Mechanical Problem	Problems associated with mechanical actions or defects, including moving parts or subassemblies, etc.
A06 – Optical Problem	Problem associated with transmission of visible light affecting the quality of the image transmitted or otherwise affecting the intended application of the visible light path.
A07 – Electrical /Electronic Property Problem	Problem associated with the function of the electrical circuitry of the device.
A08 – Calibration Problem	Problem associated with the operation of the device, related to its accuracy, and associated with the calibration of the device.
A09 – Output Problem	Problem associated with any deviation from the documented specifications of the device that relate to the end result, data, or test results provided by the device.
A10 – Temperature Problem	Problem associated with the device producing unintended temperatures
A11 – Computer Software Problem	Problem associated with written programs, codes, and/or software system that affects device performance or communication with another device.
A12 – Connection Problem	Problem associated with linking of the device and/or the functional units set up to provide means for a transfer of liquid, gas, electricity, or data.
A13 – Communication or Transmission Problem	Problem associated with the device sending or receiving signals or data. This includes transmission among internal components of the device to which the device is intended to communicate.
A14 – Infusion or Flow Problem	Problem associated with the device failing to deliver or draw liquids or gases as intended (e.g., delivering drugs at incorrect rate, problems with drawing fluid from a system). This includes vacuum collection devices and manual or mechanical pumps.
A15 – Activation, Positioning or Separation Problem	Problem associated with any deviations from the documented specifications of the device that relate to the sequence of events for activation, positioning or separation of device. Note: Deployment is synonymous with activation.
A16 – Protective Measures Problem	Problem associated with any deviations from the documented specifications of the device that relate to the implemented and inherited design features specific to devices used for reducing risks to patient or caregiver or maintaining risks within specified levels
A17 – Compatibility Problem	Problem associated with compatibility between device, patients, or substances (medication, body fluid, etc.).
A18 – Contamination /Decontamination Problem	Problem associated with the presence of any unexpected foreign substance found in the device, on its surface or in the package materials, which may affect performance or intended use of the device, or problem that compromise effective decontamination of the device.
A19 – Environmental Compatibility Problem	Problem associated with the surrounding conditions in which the device is being used such as temperature, noise, lighting, ventilation, or other external factors such as power supply.
A20 – Installation-Related Problem	Problem associated with unsatisfactory installation, configuration, and/or setup of a specific device.
A21 – Labelling, Instructions for Use or Training Problem	Problem associated with device markings/labelling, instructions for use, training and maintenance documentation, or guidelines
A22 – Human–Device Interface Problem	Problem associated with an act or omission of an act that has a different result than that intended by the manufacturer or expected by the operator
A23 – Use of Device Problem	Problem associated with failure to process, service, or operate the device according to the manufacturer’s recommendations or recognized best practices
A24 – Adverse Event Without Identified Device or Use Problem	An adverse event (e.g., patient harm) appears to have occurred, but there does not appear to have been a problem with the device or the way it was used.
A25 – No Apparent Adverse Event	A report has been received but the description provided does not appear to relate to an adverse event. This code allows a report to be recorded for administration purposes, even if it does not meet the requirements for adverse event reporting.
A26 – Insufficient Information	An adverse event appears to have occurred but there is not yet enough information available to classify the device problem.
A27 – Appropriate Term/Code Not Available	The device problem is not adequately described by any other term. Note: this code must not be used unless there is no other feasible code. The preferred term should be documented when submitting an adverse event report. This information will be used to determine if a new term should be added to the code table.

### Data collection of registries reporting TKA outliers

Outlier TKA implants currently on the market were identified by European registries publicly reporting on TKA outliers, as found in a recent systematic review [3] and non-European registries as listed on the website of the AOANJRR [19]. All available registries’ annual reports and websites were screened, and any reported TKA outlier was extracted. For all extracted TKA outliers, it was assessed whether they were reported in the latest annual reports and up-to-date website, representing TKA implants currently on the market in these registries. If the TKA outlier was not reported in the latest available registry data (i.e., not implanted in the past year in the included registries), the outlier was considered an off-market implant and excluded from further analysis.

TKA outlier definitions differed between these registries (AOANJRR: “The revision rate (per 100 component years) exceeds twice that for the group and the Poisson probability of observing that number of revisions, given the rate of the group is significant (P < 0.05)”; NJR: “having a more than twice the prosthesis time incident rate when compared to the group, allowing for confidence intervals”; SIRIS: “Revision rates of more than twice compared to the relevant group”; and the definition of an outlier was not reported for the SAR) [20].

For all TKA outliers, the year of first identification and its cumulative revision risks (1/5/10 years), including standard errors (SE) and/or 95% confidence intervals (CI), were extracted. If only the 95% CI was provided, the SE was calculated by subtracting the upper and lower bound of the 95% CI and dividing it by 3.92 [21].

### Statistics

First, the overlap between TKA implants with safety notices and TKA outliers was determined by comparing the brand name reported in both safety notices and registry data. 3 groups were distinguished: (i) TKA implants with safety notices but not identified as an outlier (“safety notices only”); (ii) TKA implants with both safety notices and identified as an outlier (“both”); (iii) TKA implants without safety notices but identified as an outlier (“outlier only”). The percentage of TKA implants in each of these groups was related to the number of unique TKA implants identified by both data sources.

Second, to prevent camouflage (i.e., multiple compatible construct combinations existing within 1 implant brand name [22]), the overlap between TKA implants with safety notices and TKA outliers across different variants/subtypes under the same brand name was analyzed. We considered possible subtypes with the same brand name by: (i) fixation (e.g., cemented versus uncemented); (ii) stability (e.g., cruciate retaining versus hinged), and (iii) mobility (e.g., fixed versus mobile).

Third, to explore possible reasons for not signaling the same TKA implants we examined: (i) differences in the frequency of IMDRF codes (Table 1) between the “safety notices only” and “both” groups, and (ii) whether the “safety notices only” group had lower cumulative revision risks (and thus seemingly better performance) than the “both” group, which may explain why they were not detected as TKA outliers. Random effects models were used to calculate the pooled cumulative revision risks (1/5/10 years) across all registries reporting on the specific TKA implant, for the “safety notices only” and “both” groups.

The metafor package in R-statistics (version 4.1.2; R Foundation for Statistical Computing, Vienna, Austria) was used for analyses.

### Ethics, registration, data sharing, use of AI, funding, and disclosures

According to Dutch law, no institutional approval was required. This work was supported by the European Union’s Horizon 2020 Research and Innovation 41 Programme (grant number 965246) and was part of the CORE-MD project. Complete disclosure of interest forms according to ICMJE are available on the article page, doi: 10.2340/17453674.2024.42361

## Results

### TKA implants with safety notices

The CORE-MD PMS tool retrieved 104,638 safety notices from 13 Ministries of Health and regulatory agencies websites, of which 1,327 safety notices were considered relevant as they matched with a specific TKA implant included in the latest annual registry reports. For the selected 1,327 safety notices, 540 safety notices were excluded because they were not related to a TKA implant (i.e., associated with surgical protocols) thus resulting in 787 safety notices included for further analysis (Figure and Supplementary Table 1). These 787 safety notices were relevant to 38 unique TKA implant brand names. Most safety notices originated from the USA and were associated with the Nexgen (Zimmer Biomet) (n = 243, 31%) (Table 2).

**Table 2:** TKA implants with the number of safety notices by country

Implant	Au	Cz	Dk	Fr	Ge	Gr	Ir	It	Ne	Sp	Sw	USA
Active Knee	1											
Advance				1				1				11
AGC Anatomic	2	1		1				1				2
Attune			1	1	3		2	2	1			20
Balansys	1	1		1	3			2				
Columbus	1				1							
Duracon	1		1					1				3
EFK					1							
Endo-Model			1	1	4		1	2		2	1	4
Evolution					2			1				3
Gemini							1			1		
Genesis	2	1		1	11	1		3	1	2		16
GMK					1		2	3				3
Innex		1			7		1	2	1	1		
iTotal					1							8
Journey	3		1	1	2			1	1	1		4
Kinemax												1
K-mod										1		
LCS					4		2	3				32
Legion					6			1	1			23
METS Smiles												1
MRK									1			
Multigen		1		1				1		1		
Mutars	1				1							
Natural-knee												1
Nexgen	2		5	1	14		8	5	1		1	206
Noiles			1		1		2	1				6
Optetrak	2					1			1			47
Persona	2		1		5			8				24
PFC Sigma					5		2	2				21
Physica								2				
Saiph	1											
Score	1				1							
Scorpio		1			4		4	2	1			23
TC-plus					1							
Triathlon	6				5		2	6		1		32
Unity					1							4
Vanguard	3	1			3	1		1	3			45

Au = Australia, Cz = Czechia, Dk = Denmark, Fr = France, Ge = Germany, Gr = Greece, Ir = Ireland, It = Italy, Ne = The Netherlands, Sp = Spain, Sw = Sweden, USA = United States of America

**Figure:**
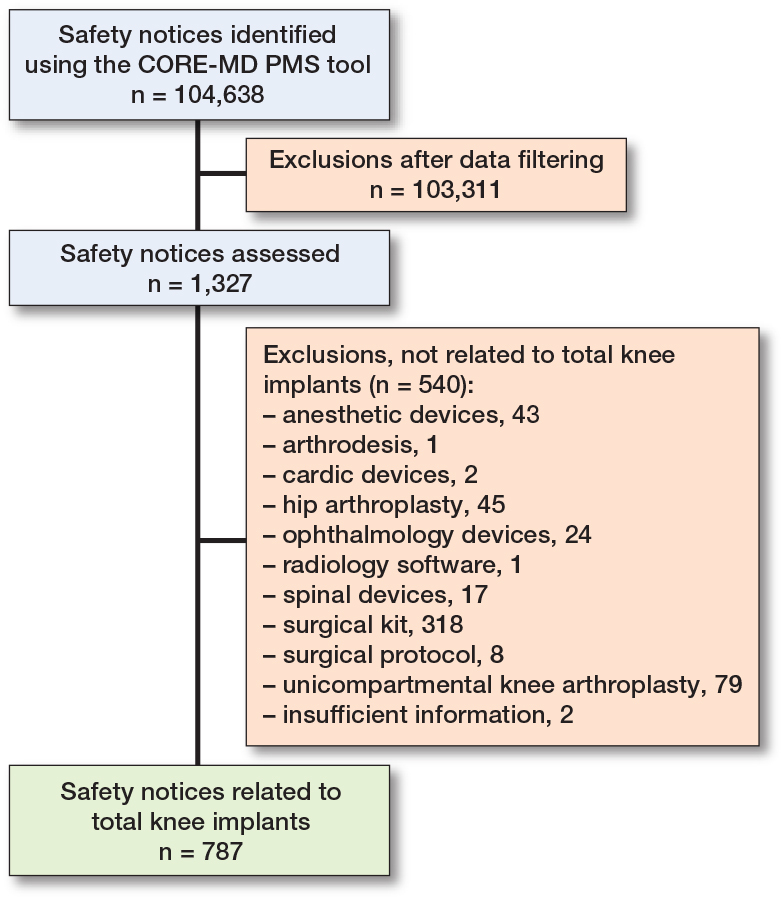
Flowchart showing the selection process of TKA implants with safety notices.

### Outlier TKA implants

4 national registries (AOANJRR, NJR, Swedish Arthroplasty Register [SAR], and SIRIS) publicly reported TKA outliers while others might report them on a secure website [3]. After removing duplicate TKA outlier brand names (i.e., the same brand name was mentioned in multiple annual reports) and off-market TKA outliers, 35 unique TKA outlier brand names were included for further analysis (Table 3). Supplementary Table 2 gives more detailed information on specific subtypes within a brand that were identified as outlier along with their performance.

**Table 3:** Outlier total knee implants currently used on the market

Outlier TKA implant	Outlier reported	TKA implants implanted	Identified by the CORE-MD PMS tool (number of safety notices)
ACS	AOANJRR, NJR	2,900	No
Active Knee	AOANJRR	7,215	Yes (1)
Advance	AOANJRR	1,009	Yes (12)
AGC Anatomic	SAR	Unknown	Yes (7)
Apex Knee	AOANJRR	513	No
Attune	AOANJRR	854	Yes (30)
Columbus	AOANJRR	6,334	Yes (2)
Duracon	SAR	Unknown	Yes (6)
E.Motion	AOANJRR, NJR,	Unknown	No
	SIRIS	(AOANJRR: 1,014, NJR: 339)	
Endo-Model	NJR	309	Yes (16)
Gemini	AOANJRR	21	Yes (2)
Genesis	AOANJRR, NJR,	Unknown	Yes (38)
	SAR	(AOANJRR: 826, NJR: 9,190)	
Journey	AOANJRR, NJR,	Unknown	Yes (14)
	SAR, SIRIS	(AOANJRR: 3,033, NJR: 1,714)	
Kinemax	SAR	Unknown	Yes (1)
LCS	AOANJRR, NJR	5,729	Yes (41)
Legion	AOANJRR, SAR	Unknown	Yes (31)
		(AOANJRR: 1,017)	
Maxim	AOANJRR	413	No
METS Smiles	NJR	954	Yes (1)
Miller-Galante	SAR	Unknown	No
Mutars	AOANJRR	357	Yes (2)
Nexgen	AOANJRR, SAR	Unknown	Yes (243)
		(AOANJRR: 2,110)	
Noiles	NJR	594	Yes (11)
Optetrak	AOANJRR, NJR	4,098	Yes (51)
Persona	SAR	Unknown	Yes (40)
PFC Sigma	AOANJRR, SAR	Unknown	Yes (30)
		(AOANJRR: 316)	
Physica	SIRIS	Unknown	Yes (2)
Profix	AOANJRR, SAR	Unknown	No
		(AOANJRR: 1,895)	
Rotaglide Plus	AOANJRR	631	No
Score	AOANJRR	4,686	Yes (2)
Scorpio	AOANJRR	1,172	Yes (35)
TC-plus	AOANJRR	63	Yes (1)
Trekking	AOANJRR	1,263	No
Triathlon	SAR	Unknown	Yes (52)
Vanguard	AOANJRR, SAR	Unknown	Yes (57)
		(AOANJRR: 6,225)	

TKA = total knee arthroplasty; CORE-MD = Coordinating Research and Evidence for Medical Devices; PMS = post-market surveillance; AOANJRR = Australian Orthopaedic Association National Joint Replacement Registry, SIRIS = Swiss National Hip & Knee Joint Registry, SAR = Swedish Arthroplasty Register, NJR = National Joint Registry for England, Wales, Northern Ireland, the Isle of Man and Guernsey.

### Overlap between TKA outliers and TKA implants with safety notices

Combining the brand names of the 38 TKA implants with a safety notice and the 35 TKA outliers resulted in 47 unique TKA implant brand names, of which 26 (55%) were in the “both” group, 12 (26%) in the “safety notices only” group, and 9 (19%) in the “outlier only” group (Tables 3 and 4). Thus, safety notices did not signal 9 (26%) of the 35 TKA outliers and registries did not signal 12 (32%) of the 38 TKA implants that had safety notices.

**Table 4:** Overlap of total knee implants in the “both” group based on fixation, mobility, and stability

Implant name	Fixation Cemented		Stability			Mobility		
	Yes	No	Cruciate retaining	Hinged	Posterior stabilized	Fixed	Mobile	Rotating
Active Knee	No	Yes	–	–	–	–	–	–
Advance	–	–	–	–	–	–	–	–
AGC Anatomic	No	–	–	–	No	No	–	–
Attune	Yes	Yes	No	–	Yes	No	–	Yes
Columbus	–	Yes	–	–	–	–	–	–
Duracon	No	–	–	–	No	–	–	–
Endo-Model	No	No	–	Yes	No	–	No	Yes
Gemini	–	–	–	–	–	–	–	Yes
Genesis	Yes	Yes	Yes	–	Yes	–	No	–
Journey	No	–	–	–	–	–	–	–
Kinemax	No	–	–	–	No	–	–	No
LCS	No	–	–	–	Yes	No	No	Yes
Legion	No	–	Yes	No	Yes	–	–	–
METS Smiles	–	–	–	–	–	–	–	–
Mutars	–	–	–	–	–	–	–	–
Nexgen	No	Yes	No	No	Yes	Yes	No	Yes
Noiles	No	–	–	Yes	–	–	–	No
Optetrak	Yes	–	–	–	Yes	–	–	–
Persona	No	No	No	–	No	–	–	–
PFC Sigma	Yes	Yes	–	–	Yes	–	Yes	–
Physica	–	–	–	–	No	–	–	–
Score	No	No	–	–	–	–	No	–
Scorpio	No	–	No	–	Yes	–	–	–
TC-plus	–	–	–	–	–	–	–	–
Triathlon	No	–	No	–	No	–	–	–
Vanguard	–	–	–	–	–	–	–	–

‘–’ = Total knee implants without any information reported in the safety notice on specific fixation/stability/mobility methods.

Yes = Total knee implants with information in the safety notice about its fixation, stability, or mobility, thus overlapping with an outlier implant based on its implant characteristic (fixation/stability/mobility).

No = Total knee implants with information in the safety notice about its fixation, stability, or mobility method but without overlapping with an outlier implant based on its implant characteristics (fixation/stability/mobility).

Considering the 26 TKA implants in the “both” group, 7 (27%) TKA implants did not have any information in the safety notice regarding fixation, 10 (38%) had no information regarding stability, and 15 (57%) no information regarding mobility, which would be needed to determine whether the exact same TKA implant was concerned (white color, Table 4). Focusing on fixation, 4 out of 26 (15%) TKA implants could be matched to the cemented subtype and 6 (23%) to the uncemented subtype (Table 4). With regard to stability, 2 out of 26 (8%) related to the cruciate retaining, 2 (8%) to the hinged, and 8 (31%) to the posterior stabilized subtype. For mobility, 1 (4%) signaled the fixed, 1 (4%) the mobile, and 5 (19%) the rotating subtype. However, 14 (54%) cemented and 3 (12%) uncemented TKA implants did not relate to the same fixation subtype (Table 4). Similarly, 6 (23%) cruciate retaining, 2 (8%) hinged, and 7 (27%) posterior stabilized TKA implants did not have the same stability and 3 (12%) fixed, 5 (19%) mobile, and 2 (8%) rotating TKA implants did not relate to the same mobility subtype.

### Revision rates, timing of safety concerns, and implant problems

For the “both” group, the median 1/5/10-year cumulative revision risks were 1.6% (range: 0.9–9.5), 6.3 (range: 3.6–23.8), and 8.2% (range: 5.6–23.8), respectively, compared with 0.6% (range: 0.3–1.1), 2.3% (range: 1.4–3.7), and 3.8% (range: 3.1–5.1), for the “safety notices only” group (Table 5).

**Table 5:** Total knee implants brand names with at least 1 safety notice

Implant	Date of first safety notice	Identified as outlier	Year first identified as outlier and registry reporting	Pooled cumulative revision risk (CI) for specified implant brand name		
				1-year	5-year	10-year
Active Knee	21/10/2016	Yes	2016 (AOANJRR)	1.1 (0.9–1.4) **^a^**	5.0 (4.6–5.6) **^a^**	8.8 (8.1–9.5) **^a^**
Advance	11/7/2016	Yes	2013 (AOANJRR)	2.0 (1.3–3.1) **^a^**	6.4 (5.0–8.2) **^a^**	8.1 (6.4–10.2) **^a^**
AGC Anatomic	21/7/2015	Yes	2014 (SAR)	–	–	–
Attune	29/6/2015	Yes	2023 (AOANJRR)	1.8 (1.0–3.0) **^a^**	–	–
Balansys	29/1/2014	No	–	0.9 (0.5–1.2) **^a,d,e,f^**	3.1 (2.3–3.9) **^a,c,d,e,f^**	5.1 (2.2–8.1) **^a,d^**
Columbus	17/1/2008	Yes	2009 (AOANJRR)	1.2 (0.9–1.5) **^a^**	4.4 (3.7–5.3) **^a^**	7.3 (6.0–8.8) **^a^**
Duracon	20/9/2007	Yes	2004 (SAR)	–	–	–
EFK	15/4/2014	No	–	0.6 (0.1–1.2) **^f^**	1.7 (0.5–3.0) **^f^**	–
Endo–Model	16/4/2012	Yes	2019 (NJR)	1.3 (0.8–2.2) **^b^**	4.8 (3.7–6.3) **^b^**	7.0 (5.3–9.2) **^b^**
Evolution	17/2/2015	No	–	0.7 (0.3–1.1) **^a,b,f,g^**	2.8 (2.1–3.5) **^a,b,g^**	–
Gemini	7/9/2010	Yes	2007 (AOANJRR)	9.5 (2.5–33.0) **^a^**	23.8 (10.7–48.1) **^a^**	23.8 (10.7–48.1) **^a^**
Genesis	9/5/2006	Yes	2004 (AOANJRR),	1.0 (0.7–1.3) **^a,b^**	3.6 (3.2–4.1) **^a,b^**	5.6 (4.8–6.3) **^a,b^**
			2018 (SAR), 2021 (NJR)			
GMK Sphere	3/7/2017	No	–	1.1 (0.9–1.4) **^a,b,e,f,g^**	3.7 (2.9–4.5) **^a,b,e,g^**	4.3 (2.4–6.1) **^a^**
Innex	25/7/2005	No	–	0.9 (0.5–1.3) **^d,e,f^**	2.8 (2.0–3.6) **^c,d,e,f^**	3.5 (2.4–4.6) **^d^**
iTotal	23/7/2012	No	–	0.4 (0.2–0.9) **^e^**	3.5 (2.5–5.0) **^e^**	–
Journey	3/1/2014	Yes	2009 (AOANJRR), 2018 (SAR),	1.6 (0.1–3.1) **^a,b,e^**	6.3 (1.8–10.8) **^a,b,e^**	11.0 (9.9–12.2) **^a^**
			2019 (SIRIS), 2014 (NJR)			
Kinemax	14/5/2015	Yes	2006 (SAR)	–	–	–
K-mod	19/5/2021	No	–	–	–	–
LCS	2/12/2005	Yes	2012 (AOANJRR), 2021 (NJR)	0.9 (0.2–1.6) **^a,b^**	5.6 (1.8–9.5) **^a,b^**	7.7 (2.5–12.8) **^a,b^**
Legion	22/8/2009	Yes	2017 (AOANJRR), 2019 (SAR)	3.3 (2.3–4.6) **^a^**	6.3 (4.8–8.3) **^a^**	9.9 (7.5–13.0) **^a^**
METS Smiles	17/8/2016	Yes	2018 (NJR)	–	–	–
MRK	31/12/2021	No	–	0.3 (0.0–0.6) **^a,b,d^**	1.8 (1.2–2.3) **^a,b,d^**	3.1 (1.6–4.6) **^a,b^**
Multigen	12/5/2021	No	–	–	–	–
Mutars	3/4/2013	Yes	2023 (AOANJRR)	6.5 (4.2–9.9) **^a^**	–	–
Natural-knee	7/11/2019	No	–	0.4 (0.2–0.7) **^a,d,f,g^**	1.7 (1.2–2.1) **^a,d,f,g^**	3.2 (2.4–3.9) **^a,d^**
Nexgen	13/9/2004	Yes	2018 (AOANJRR), 2015 (SAR)	2.4 (1.9–3.2) **^a^**	5.0 (4.2–6.1) **^a^**	6.9 (5.1–9.2) **^a^**
Noiles	2/3/2014	Yes	2018 (NJR)	–	–	–
Optetrak	1/6/2006	Yes	2007 (AOANJRR)	1.0 (0.0–2.1) **^a^**	10.3 (4.1–16.4) **^a^**	13.7 (7.0–20.4) **^a^**
Persona	21/11/2012	Yes	2021 (SAR)	–	–	–
PFC Sigma	2/12/2005	Yes	2018 (AOANJRR), 2012 (SAR)	2.2 (1.1–4.6) **^a^**	7.1 (4.7–10.5) **^a^**	7.4 (5.0–10.9) **^a^**
Physica	18/4/2019	Yes	2019 (SIRIS)	1.7 (1.3–2.3) **^e^**	6.8 (5.9–7.9) **^e^**	–
Saiph	25/3/2022	No	–	0.6 (0,3–1,0) **^b^**	1,4 (0,9–2,0) **^b^**	–
Score	4/10/2019	Yes	2013 (AOANJRR)	1.5 (0.8–2.2) **^a^**	6.5 (5.5–7.6) **^a^**	11.1 (9.3–12.8) **^a^**
Scorpio	26/8/2005	Yes	2014 (AOANJRR)	1.2 (0.7–2.0) **^a^**	6.1 (4.9–7.7) **^a^**	7.4 (6.0–9.2) **^a^**
TC-plus	10/6/2008	Yes	2008 (AOANJRR)	1.6 (0.2–10.7) **^a^**	8.4 (3.6–19.1) **^a^**	14.4 (7.4–26.9) **^a^**
Triathlon	7/2/2007	Yes	2021 (SAR)	–	–	–
Unity	30/9/2021	No	–	0.4 (0.1–0.9) **^a,b,f^**	1.5 (0.7–2.3) **^b,f^**	–
Vanguard	17/11/2016	Yes	2012 (AOANJRR), 2009 (SAR)	1.9 (1.2–2.6) **^a^**	5.9 (4.7–7.1) **^a^**	8.2 (6.8–9.5) **^a^**

a Based on revision risks as reported by the AOANJRR;

b based on revision risks as reported by the NJR;

c based on revision risks as reported by the RIPO;

d based on revision risks as reported by the LROI;

e based on revision risks as reported by the SIRIS;

f based on revision risks as reported by the EPRD;

g based on revision risks as reported by the AJRR.

TKA = total knee arthroplasty; CI = confidence intervals; AOANJRR = Australian Orthopaedic Association National Joint Replacement Registry; SIRIS = Swiss National Hip & Knee Joint Registry; SAR = Swedish Arthroplasty Register; NJR = National Joint Registry for England; Wales, Northern Ireland, the Isle of Man and Guernsey.

When comparing the dates of the first issuance of safety notices with the dates when the implant was first identified as outlier by registries, no specific data source consistently published safety signals earlier (Table 5).

For the 26 TKA implants in the “both” group, 728 safety notices were issued with the most frequently reported problem being related to “A02–Manufacturing, Packaging or Shipping” (43%), followed by “A23–Use of Device” (16%) (Table 6). The most frequent type of problem found was similar for the 12 TKA implants in the “safety notices only” group (n = 59 safety notices): “A02–Manufacturing, Packaging or Shipping” (44%) (Table 6). Focusing on differences between the 2 groups, safety notices related to “A05–Mechanical Problem” (6%) and “A17–Compatibility Problem” (8%), respectively, were reported only for the “both” group (Table 6) but not encountered for the “safety notices only” group (Table 6). The interobserver agreement to classify safety notices according to the IMDRF codes among the 2 observers was substantial (κ = 0.79; CI 0.76–0.82).

**Table 6:** IMDRF medical device problem codes described in safety notices. Values are count (%)

IMDRF code	TKA implants (n = 26) in the “both” group	TKA implants (n = 12) in the “safety notices only” group
A01	–	4 (6.7)
A02	313 (43)	26 (44)
A04	56 (7.7)	7 (12)
A05	41 (5.6)	–
A09	6 (0.8)	–
A17	59 (8.1)	–
A18	9 (1.2)	1 (1.7)
A20	2 (0.3)	–
A21	70 (10)	11 (19)
A23	113 (16)	6 (10)
A24	34 (4.7)	1 (1.7)
A26	25 (3.4)	3 (5.1)
Total	728	59

## Discussion

Our study is the first to assess the extent of overlap in TKA implants for which safety notices were issued and that were identified as outliers in registry data. We aimed to assess the extent to which safety notices and outlier identification in registries signal the same or different TKA implants. We found that approximately half (55%) of the TKA implants were identified by both safety notices and registries outlier identification procedures, but a quarter of the TKA outliers did not have any publicly released safety notices on the websites of Ministries of Health or regulatory agencies. In addition, there were implant problems identified by safety notices that did not manifest in an outlier status. TKA implants with both safety notices and an outlier status had higher cumulative revision risks (1/5/10 years) than TKA implants with safety notices only.

A recent review that assessed the current state of medical device safety signal detection stated that a global dataset of medical devices should be created using automatic reports from national/regional databases [23]. In the absence of such a global dataset, the CORE-MD PMS tool was developed recently [7]. Our results add that such a global dataset of safety notices may still not identify a quarter of TKA implants with statistically significant poor performance (i.e., TKA outliers). Additionally, a published safety notice by itself does not constitute a sufficient and necessary condition for being identified as a TKA outlier (the “safety notices only” group). We identified that certain IMDRF codes “A05–Mechanical Problem” and “A17–Compatibility Problem” were not encountered in the “safety notices only” group, suggesting that these are more closely related to poorer implant performance. This observation could result in a helpful indication to highlight a higher risk for certain TKA implants with such IMRDF codes identified in safety notices to become an outlier, thus warranting closer scrutiny of these TKA implants.

This multi-registry analysis examined the content of safety notice text, which does not typically include information needed to identify specific variants/subtypes of TKA implants, characterized by fixation, stability, and mobility. Such a lack of information causes camouflage (i.e., multiple implant subtypes exist under the same implant brand name) [22] making it difficult or even impossible to link the correct TKA implants with safety notices to registry data, or to combine data from different real-world data sources. This information is, however, important for action to be taken, as illustrated by a recent study showing good performance for the Nexgen system but significantly higher revision risks for specific combinations with the Nexgen LPS Flex (see also Supplementary Table 2) [24]. In addition, registries often publicly report only TKA implants’ brand names without listing more detailed information (e.g., fixation, stabilization, and mobility) to identify which specific subtype of an implant is concerned. Product codes and unique device identifiers (UDIs), which would be needed to deal with such camouflage, were also not reported in safety notices or publicly by registries, except for the American medical device recall database. Accordingly, we highly recommend minimal reporting requirements for manufacturers with respect to safety notices and also for registries when reporting outliers, including: full brand name, fixation, mobility, stability, and product codes or UDIs.

Arthroplasty registries currently only identify TKA outliers based on revision risks, which may take several years (at least 1) before sufficient numbers are available to detect performance problems [3,4]. Using revision risk may seem a relatively straightforward endpoint (the occurrence of revision surgery), but surgeon, implant, and patient factors determine whether an implant is revised. Moreover, between-registry variation exists regarding definitions and reasons for revision [3,25] although all included registries identifying TKA outliers defined revision as “the replacement/removal/addition of one or more prosthetic components”. But, for instance, revisions due to infection are excluded from the all-cause revision risk in the Swedish registry [26,27]. In contrast, the NJR also includes revision due to infection if no prosthetic component was exchanged, which can result in specific TKA implants being identified as an outlier in the NJR but not in other registries. Interestingly, the number of TKA outliers publicly reported by the AOANJRR is much higher when compared with other registries publicly reporting on outliers. Part of the explanation may be related to the definition, such as the minimum number of implants required for the publication and analysis of implant-specific revision rates, which is much lower in the AOANJRR (500 procedures compared with 2,500 procedures required in the NJR). These heterogeneities highlight the importance of an international agreement on definitions and outcomes, as well as time-points and methodology used for measuring outcomes within registries.

Some safety notices may be released based on implant-related problems causing clinical performance issues relating to a specific TKA implant but also on a case-by-case analysis (i.e., no minimum number of implants at risk is required), meaning that safety notices may provide an earlier signal of a possible performance problem than registries [28]. Accordingly, registries could use this as a signal to analyze specific TKA implants with released safety notices to detect potential adverse trends in performance earlier. However, when considering the timing of safety notices and outlier data being published, none of the data sources consistently released safety signals earlier than any other, highlighting the importance of a multifaced approach combining these 2 data sources. While this provides relevant information and includes all TKA implants for which safety concerns were reported in safety notices or reported as an outlier, it does not answer the question as to what percentage of TKA implants did not have any safety concerns reported. It would seem rather infeasible to estimate this percentage based on all TKA implants currently on the market in all countries examined in the present study. Creating a random sample of TKA implants would be a more feasible alternative to provide such information as a next step.

### Limitations

First, the CORE-MD PMS tool searched for safety notices published on the websites of Ministries of Health and regulatory agencies, but we may have missed safety notices if these were reported only on manufacturers’ websites, which would have underestimated the number of TKA outliers with safety notices. Second, both TKA outliers and TKA implants not identified as a TKA outlier had a relatively similar distribution of IMDRF-problem types, suggesting that the IMDRF code may not be sufficient to distinguish between these 2 groups. However, only the Level 1 IMDRF codes were used due to the large number of safety notices to be manually classified, so there may be differences in distribution when Level 2 or 3 problem terms were used. On the other hand, one could argue that such differences in these more detailed problem-type descriptions would not likely entail clinically relevant differences in problems. Third, other factors such as surgeon or hospital performance are known to influence revisions, which may skew the revision risks data. Nonetheless, as we used data from 4 national registries consisting of a large number of TKA outliers, the impact on our results is likely to have been small. Fourth, safety notices were collected from websites in more countries than those for which registry outlier identification data were available, which might have underestimated the number of TKA outliers and explain part of the “safety notices only” group. On the other hand, assuming that safety notices point to a problem with the implant itself, we would expect any performance issue to be similar across countries and thereby picked up by other registries as well. Finally, our analysis does not exclude possible duplicates of the same safety notices published in different countries or for different models/lots within the country. This is because different countries use diverse formats and criteria to issue safety notices: some countries issue separate safety notices for each model (e.g., the USA, resulting in a high number of safety notices from the USA), while others publish only 1 safety notice with multiple models. However, the safety notices would still signal the same TKA implant, which was used as the unit of analysis in the present study, so excluding duplicate safety notices would not have changed our results.

### Conclusion

We found that approximately half (55%) of the TKA implants were identified by both safety notices and registries outlier identification procedures, whereas around 25% of TKA outliers are not the subject of publicly released safety notices, highlighting the potential of adopting a multifaceted approach, integrating various real-world data sources and methods to combine information to enhance medical device safety signal detection.

### Supplementary data

Supplementary Tables 1–2 are available as Suppldata on the article page, doi: 10.2340/17453674.2024.42361

## Supplementary Material



## Notes

LH, YR: data collection, statistical analysis, interpretation of data, writing the original draft of the manuscript. AAS-P, AGF, AL, EGC, REG, RGHHN, and TM: critical revision of the manuscript for important intellectual content. PJM-vdM: interpretation of data, critical revision of the manuscript for important intellectual content.

Handling co-editors: Keijo Mäkelä and Robin Christensen

*Acta* thanks Gary Hooper and Einar Andreas Sivertsens for help with peer review of this manuscript.
