# Supplementary material for: Safety notices and registry outlier data measure different aspects of safety and performance of total knee implants: a comparative study of safety notices and register outliers
Source: Acta Orthop. 2024 Nov 25;95:667–76. doi: 10.2340/17453674.2024.42361 (PMC11587162; doi:10.2340/17453674.2024.42361)
Supplement: Supplementary file 1 [file ActaO-95-42361-s1.pdf]

Supplementary Table 1: Countries included in the CORE-MD PMS tool				
**Country**	**Last update date**	**Safety notices (n)**	**Safety notices selected (n)**	**Safety notices on TK implants (n)**
Australia (SARA)	31/05/2023	7,208	53	29
Czechia	30/03/2023	3,135	15	7
Denmark	30/03/2023	4,652	22	11
France	12/04/2023	1,474	20	8
Germany	30/03/2023	14,544	192	87
Greece	13/04/2023	885	5	3
Ireland	31/03/2023	7,096	63	27
Italy	29/03/2023	8,713	92	51
Portugal	13/04/2023	67	0	0
Spain	12/04/2023	3,593	25	10
Sweden	12/04/2023	679	2	2
The Netherlands	31/03/2023	3,830	35	12
The USA (Medical Device Recall Database)	01/04/2023	48,762	803	540
Total amount		104,638	1,327	787

Supplementary Table 2. Detailed information of outlier TKs implants currently used on the market

Brand name and subtype of outlier TK–implant (femoral–tibial component)	Outlier reported in registry				Outlier revision (latest available revision data)
	AOANJRR	NJR	SAR	SIRIS	
**ACS**<br>ACS/ACS Mobile PC (uncemented)	2016–2023	–	–	–	Revision/100 observed years:<br>3.03 (CI 2.04–4.32) *versus* other TKs 0.53 (CI 0.52–0.54)
ACS (uncemented)/ACS Fixed	2016–2023	–	–	–	Revision/100 observed years:<br>0.84 (CI 0.70–1.00) *versus* other TKs 0.53 (CI 0.52–0.53)
ACS/ACS Fixed	2015	–	–	–	Revision/100 observed years: 2.11
ACS/ACS	2013, 2014	–	–	–	Revision/100 observed years: 2.37
ACS/ACS Mobile	2015	–	–	–	Revision/100 observed years: 2.06
ACS	–	2017	–	–	Prosthesis Time Incident Rate: 1.47
**Active Knee**<br>Active Knee (uncemented)/Active Knee	2016–2023	–	–	–	Revision/100 observed years:<br>0.91 (CI 0.85–0.98) *versus* other TKs 0.52 (CI 0.52–0.53)
**Advance**<br>Advance/Advance	2013–2023	–	–	–	Revision/100 observed years:<br>0.94 (CI 0.74–1.18) *versus* other TKs 0.53 (CI 0.52–0.54)
**AGC Anatomic**	–	–	2014[b,c,e,], 2015[a,b,c,e,], 2016[a,b,c,e,], 2017[a,b,c,e,], 2018[a,b,c,e,], 2019[b,e,], 2020[b,e,]	–	[b] Risk of revision (RR): 1.41 (CI 1.04–1.90)<br>[e] RR: 1.56 (CI 1.13–2.14)
**Apex Knee**<br>Apex Knee CR (uncemented)/Apex Knee (uncemented)	2018–2023	–	–	–	Revision/100 observed years:<br>0.86 (CI 0.57–1.24) *versus* other TKs 0.53 (CI 0.52–0.54)
**Attune**<br>Attune PS (cemented)/Attune (uncemented)	2023	–	–	–	Revision/100 observed years:<br>2.08 (CI 1.28–3.17) *versus* other TKs 0.53 (CI 0.52–0.54)

**Columbus**<br>Columbus/Columbus	2009–2023	–	–	–	Revision/100 observed years:<br>0.82 (CI 0.71–0.94) *versus* other TKs<br>0.53 (CI 0.52–0.53)
**Duracon**	–	–	2004[d], 2012[d], 2013[d,f], 2015[a,b,], 2016[a,b,c,e,], 2017[a,b,e,], 2018[a,b,c,e,]	–	[a] RR: 1.34 (CI 1.06–1.70)<br>[b] RR: 1.45 (CI 1.11–1.88)<br>[c] RR: 1.30 (CI 1.00–1.70)<br>[e] RR: 1.38 (CI 1.03–1.85)
**E.Motion**<br>E.motion PS	–	–	–	2020	Revised/total included in evaluation = 19/312
E–Motion Bicondylar Knee with primary patella	–	2020	–	–	Prosthesis Time Incident Rate: 1.13
E.Motion/E.Motion	2012–2023	–	–	–	Revision/100 observed years:<br>0.96 (CI 0.75–1.21) *versus* other TKs<br>0.53 (CI 0.52–0.54)
**Endo–Model**<br>Endo–Model Modular Rotating Hinge	–	2019	–	–	Prosthesis Time Incident Rate: 1.90
**Gemini**<br>Gemini MK II/ Gemini MK II	2007–2023	–	–	–	Revision/100 observed years:<br>3.64 (CI 1.57–7.16) *versus* other TKs<br>0.53 (CI 0.52–0.54)
**Genesis**<br>Genesis II/Mobile Bearing	2004	–	–	–	Hazard ratio (HR) (adjusted for age and sex, *versus* Genesis II/Genesis II): 2.57 (CI 1.74–3.79)
Genesis (cemented)/Genesis (cemented)	2010–2023	–	–	–	Revision/100 observed years:<br>1.55 (CI 0.77–2.78) *versus* other TKs<br>0.53 (CI 0.52–0.54)
Genesis II Oxinium PS (cemented)/Genesis II (keel)	2009–2023	–	–	–	Revision/100 observed years:<br>2.26 (CI 1.76–2.86) *versus* other TKs<br>0.53 (CI 0.52–0.53)
Genesis II Oxinium CR (uncemented)/Genesis II	2014–2023	–	–	–	Revision/100 observed years:<br>4.59 (CI 3.37–6.10) *versus* other TKs<br>0.53 (CI 0.52–0.54)
Genesis II/Mobile bearing with Oxinium femoral component (uncemented)	2005, 2006	–	–	–	Revision/100 observed years:<br>27.0 (CI 19.71–36.16)

Genesis II Oxinium (uncemented)/Profix Mobile	2013	–	–	–	Revision/100 observed years: 12.94
Genesis II CR (uncemented)/Profix Mobile (cemented)	2014–2023	–	–	–	Revision/100 observed years: 1.25 (CI 0.88–1.72) *versus* other TKs 0.53 (CI 0.52–0.54)
Genesis II Oxinium PS (cemented)/Genesis II (uncemented)	2014–2023	–	–	–	Revision/100 observed years: 3.39 (CI 1.98–5.43) *versus* other TKs 0.53 (CI 0.52–0.54)
Genesis II Oxinium CR (uncemented)/Profix Mobile	2014–2023	–	–	–	Revision/100 observed years: 8.96 (CI 6.78–11.61) *versus* other TKs 0.53 (CI 0.52–0.54)
Genesis II/Fixed bearing with Oxinium femoral component (uncemented)	2005, 2006	–	–	–	Revision/100 observed years: 17.6 (CI 12.53–24.10)
Genesis II Oxinium (uncemented)/Genesis II	2007–2013	–	–	–	Revision/100 observed years: 6.53
Genesis II CR (uncemented)/Genesis II (uncemented)	2010–2014	–	–	–	Revision/100 observed years: 1.51
Genesis II Oxinium (uncemented)/MBK	2007–2012	–	–	–	Revision/100 observed years: 14.00
Genesis II Oxinium PS (cemented)/Genesis II	2008	–	–	–	Revision/100 observed years: 1.63
Genesis II	–	–	2018[d]	–	[d] RR: 4.89 (CI 1.12–21.23)
Genesis II/Legion	–	–	2019[a,b,c,e], 2020[a,b,c,e],	–	[a] RR: 1.67 (CI 1.18–2.37)<br>[b] RR: 2.08 (CI 1.38–3.13)<br>[c] RR: 1.69 (CI 1.18–2.42)<br>[e] RR: 2.22 (CI 1.47–3.33)
Legion/Genesis II Pri MBT	–	–	2021[a,b,c], 2022[a,b,c]	–	[a] HR: 1.88 (CI 1.44–2.47)<br>[c] HR: 1.66 (CI 1.29–2.13)
Genesis II Oxinium PS	–	2021	–	–	Prosthesis Time Incident Rate: 0.86
Genesis II Oxinium without primary patella	–	2021	–	–	Prosthesis Time Incident Rate: 0.78
**Journey**<br>Journey Oxinium/Journey	2015–2023	–	–	–	Revision/100 observed years: 1.20 (CI 1.08–1.32) *versus* other TKs 0.53 (CI 0.52–0.54)

Journey/Journey	2009–2014	–	–	–	Revision/100 observed years: 1.41
Journey II	–	–	–	2019–2023	Risk-adjusted HR for 2-year revision risk (adjusted for age/sex/BMI/ASA/ Charnley class): 1.81 (CI 1.48–2.20) and (adjusted for age and sex): 1.93 (CI 1.64–2.29)
Journey	–	–	2018[a,b,c,e], 2019[a,b,c,e], 2020[a,b,c,e], 2021[a,b,c,d,e,f,g,h], 2022[a,b]	–	[a] HR: 4.24 (CI 2.54–7.08)<br>[b] HR: 5.16 (CI 3.03–8.80)
Journey Deuce	–	2014	–	–	Prosthesis Time Incident Rate: 2.42
Journey Oxinium	–	2017	–	–	Prosthesis Time Incident Rate: 0.94
Journey II BCS Oxinium without primary patella	–	2020	–	–	Prosthesis Time Incident Rate: 1.30
**Kinemax**	–	–	2006[d], 2007[a,d,g], 2008[a,d,c], 2009[a,d.c], 2010[a,b,c,d,e,f], 2011[a,b,c,d,e,f,g,h], 2012[a,b,Ωf,g,h]	–	[a] RR: 1.72 (CI 1.27–2.33)<br>[b] RR: 1.60 (CI 1.18–2.17)<br>[d] RR: 3.26 (CI 1.82–5.84)<br>[f] RR: 3.16 (CI 1.76–5.65)<br>[g] RR: 4.57 (CI 1.63–12.83)<br>[h] RR: 4.37 (CI 1.56–12.29)
**LCS**<br>LCS PS	2014–2023	–	–	–	Revision/100 observed years: 1.22 (CI 0.96–1.53) *versus* other TKs 0.53 (CI 0.52–0.54)
LCS Duofix	2012–2023	–	–	–	Revision/100 observed years: 1.21 (CI 1.12–1.31) *versus* other TKs 0.53 (CI 0.52–0.54)
LCS PFJ	–	2021	–	–	Prosthesis Time Incident Rate: 4.65
**Legion**<br>Legion Revision Tibial Baseplate	2017–2023	–	–	–	Revision/100 observed years: 1.24 (CI 0.96–1.57) *versus* other TKs 0.53 (CI 0.52–0.53)
Legion/Genesis II Pri MBT	–	–	2021[a,b,c], 2022[a,b,c]	–	[a] HR: 1.88 (CI 1.44–2.47)

					[c] HR: 1.66 (CI 1.29–2.13)
Genesis II/Legion	–	–	2019[a,b,c,e], 2020[a,b,c,e]	–	[a] RR: 1.67 (CI 1.18–2.37)<br>[b] RR: 2.08 (CI 1.38–3.13)<br>[c] RR: 1.69 (CI 1.18–2.42)<br>[e] RR: 2.22 (CI 1.47–3.33)
Legion Oxinium FS	2022–2023	–	–	–	Revision/100 observed years:<br>1.52 (CI 1.08–2.08) *versus* other TKs<br>0.53 (CI 0.52–0.54)
**Maxim**<br>Maxim (uncemented)/Vanguard (cemented)	2020–2023	–	–	–	Revision/100 observed years:<br>1.27 (CI 0.99–1.61) *versus* other TKs<br>0.53 (CI 0.52–0.54)
Vanguard PS/Maxim	2014–2018	–	–	–	Revision/100 observed years: 1.04
**METS Smiles**<br>Smiles (METS hinged/linked knee)	–	2018	–	–	Prosthesis Time Incident Rate: 1.34
**Miller–Galante**<br>Miller–Galante II	–	–	2001[a]	–	[a] RR (CI 1.06–2.16)
Miller–Galante unspecified	–	–	2001[a], 2002[a], 2003[a]	–	[a] RR (CI 1.44–3.29)
**Mutars**	2023	–	–	–	Revision/100 observed years:<br>6.16 (CI 4.59–8.10) *versus* other TKs<br>0.53 (CI 0.52–0.53)
**Nexgen**<br>NexGen APT	–	–	2015[e], 2016[e], 2017[e], 2018[e]	–	[e] RR 1.28 (CI 1.02–1.60)
Nexgen LPS Flex (uncemented)/Nexgen	2018–2023	–	–	–	Revision/100 observed years:<br>0.81 (CI 0.66–0.97) *versus* other TKs<br>0.53 (CI 0.52–0.53)
**Noiles**	–	2018	–	–	Prosthesis Time Incident Rate: 1.19
**Optetrak**<br>Optetrak–PS/Optetrak	2007–2023	–	–	–	Revision/100 observed years:<br>1.23 (CI 1.10–1.37) *versus* other TKs<br>0.53 (CI 0.52–0.54)

Optetrak–PS/Optetrak–RBK	2008–2023	–	–	–	Revision/100 observed years:<br>0.91 (CI 0.74–1.12) *versus* other TKs<br>0.53 (CI 0.52–0.54)
Optetrak–PS/Optetrak–PS	2009–2023	–	–	–	Revision/100 observed years:<br>2.50 (CI 1.36–4.19) *versus* other TKs<br>0.53 (CI 0.52–0.54)
Optetrak–CR (cemented)/Optetrak (cemented)	2014, 2019–2023	–	–	–	Revision/100 observed years:<br>1.32 (CI 0.68–2.31) *versus* other TKs<br>0.53 (CI 0.52–0.54)
Optetrak–CR/Optetrak	2010, 2011	–	–	–	Revision/100 observed years: 1.11
Optetrak PS	–	2022	–	–	Prosthesis Time Incident Rate: 0.97
**Persona**	–	–	2021[b]	–	[b] HR: 1.66 (CI 1.00–2.75)
**PFC Sigma**<br>PFC Sigma PS (cemented)/MBT (uncemented)	2018, 2020–2023	–	–	–	Revision/100 observed years:<br>0.85 (CI 0.55–1.25) *versus* other TKs<br>0.53 (CI 0.52–0.54)
PFC–RP	–	–	2013[a,b,c,e], 2014[a,b,c,e], 2015[a,b,c,e], 2016[a,b,c,e], 2017[a,b,c,e], 2018[a,b,c,e], 2019[a,b,c,e], 2020[a,b,c,e]	–	[a] RR: 1.74 (CI 1.28–2.37)<br>[b] RR: 1.91 (CI 1.38–2.64)<br>[c] RR: 1.74 (CI 1.25–2.43)<br>[e] RR: 1.92 (CI 1.35–2.73)
PFC	–	–	2003[a], 2004[a,d], 2005[a,c,d], 2006[a,c,d], 2007[a,c,d], 2008[a,c,d], 2009[a,d]	–	[a] RR: 1.48 (CI 1.01–2.15)<br>[d] RR: 3.08 (CI 1.31–7.25)
PFC Rot. Platf.	–	–	2012[a,b,c]	–	[a] RR: 1.53 (CI 1.10–2.13)<br>[b] RR: 1.51 (CI 1.08–2.12)<br>[c] RR: 1.53 (CI 1.07–2.19)
PFC Sigma MBT	–	–	2013[d,f]	–	[d] RR: 2.65 (CI 1.54–4.56)<br>[f] RR: 2.22 (CI 1.25–3.92)
PFC Sigma TC–3 (revision)	–	–	2022[a,b]	–	[a] HR: 1.89 (CI 1.04–3.44)<br>[b] HR: 2.28 (CI 1.22–4.26)

**Physica**<br>Physica KR	–	–	–	2019–2023	Risk-adjusted HR for 2-year revision risk (adjusted for age/sex/BMI/ASA/ Charnley class): 3.06 (CI 1.14–8.17) and (adjusted for age and sex): 3.80 (CI 2.04–7.07)
Physica PS	–	–	–	2019–2023	Risk-adjusted HR for 2-year revision risk (adjusted for age/sex/BMI/ASA/ Charnley class): 2.91 (CI 1.65–5.51) and (adjusted for age and sex): 3.11 (CI 1.84–5.25)
Physica KR/PS	–	–	–	2022–2023	Risk-adjusted HR for 2-year revision risk (adjusted for age/sex/BMI/ASA/ Charnley class): 2.83 (CI 1.73–4.63) and (adjusted for age and sex): 3.25 (CI 2.17–4.85)
**Profix**<br>Profix Oxinium (uncemented)/Profix Mobile	2013–2023	–	–	–	Revision/100 observed years: 5.09 (CI 3.97–6.42) *versus* other TKs 0.53 (CI 0.52–0.54)
Profix Oxinium (uncemented)/Profix	2007–2012, 2014–2023	–	–	–	Revision/100 observed years: 4.43 (CI 3.05–6.22) *versus* other TKs 0.53 (CI 0.52–0.54)
Profix/Profix Mobile	2004, 2007–2023	–	–	–	Revision/100 observed years: 0.88 (CI 0.73–1.06) *versus* other TKs 0.53 (CI 0.52–0.54)
Profix Oxinium (cemented)/Profix (uncemented)	2018, 2020–2023	–	–	–	Revision/100 observed years: 1.08 (CI 0.59–1.81) *versus* other TKs 0.53 (CI 0.52–0.54)
Profix Oxinium (cemented)/Profix Mobile	2013–2018, 2020– 2023	–	–	–	Revision/100 observed years: 0.91 (CI 0.61–1.30) *versus* other TKs 0.53 (CI 0.52–0.54)

Profix	–	–	2011[d], 2012[d], 2013[d], 2015[e], 2016[b,e], 2017[b,e], 2018[e]	–	[e] RR: 1.43 (CI 1.04–1.97)
Profix/fixed bearing with Oxinium femoral component (uncemented)	2005, 2006	–	–	–	Revision/100 observed years: 15.2
Profix/mobile bearing with Oxinium femoral component (uncemented)	2005, 2006	–	–	–	Revision/100 observed years: 16.3
Profix Oxinium (uncemented)/MBK	2007–2012	–	–	–	Revision/100 observed years: 7.92
Profix Oxinium (cemented)/MBK	2012	–	–	–	Revision/100 observed years: 1.39
Genesis II Oxinium (uncemented)/Profix Mobile	2013	–	–	–	Revision/100 observed years: 12.94
Genesis II CR (uncemented)/Profix Mobile (cemented)	2014–2023	–	–	–	Revision/100 observed years: 1.25 (CI 0.88–1.72) *versus* other TKs 0.53 (CI 0.52–0.54)
Genesis II Oxinium CR (uncemented)/Profix Mobile	2014–2023	–	–	–	Revision/100 observed years: 8.96 (CI 6.78–11.61) *versus* other TKs 0.53 (CI 0.52–0.54)
**Rotaglide Plus**<br>Rotaglide Plus/Rotaglide Plus	2008–2023	–	–	–	Revision/100 observed years: 1.08 (CI 0.86–1.33) *versus* other TKs 0.53 (CI 0.52–0.54)
**Score**<br>Score (uncemented)/Score (cemented)	2019–2023	–	–	–	Revision/100 observed years: 1.15 (CI 0.95–1.39) *versus* other TKs 0.53 (CI 0.52–0.53)
Score (uncemented)/Score (uncemented)	2014–2023	–	–	–	Revision/100 observed years: 1.20 (CI 1.06–1.36) *versus* other TKs 0.53 (CI 0.52–0.53)
Score/Score	2013	–	–	–	Revision/100 observed years: 1.53
**Scorpio**<br>Scorpio NRG PS (uncemented)/Series 7000 (uncemented)	2014–2023	–	–	–	Revision/100 observed years: 0.78 (CI 0.63–0.96) *versus* other TKs 0.53 (CI 0.52–0.54)

**TC–plus**<br>TC–Plus (uncemented)/TC–Plus (cemented)	2018–2023	–	–	–	Revision/100 observed years:<br>1.10 (CI 0.48–2.18) *versus* other TKs<br>0.53 (CI 0.52–0.54)
TC–Plus/TC–Plus	2008–2012	–	–	–	Revision/100 observed years: 1.71
**Trekking**<br>Trekking/Trekking	2013–2023	–	–	–	Revision/100 observed years:<br>0.86 (CI 0.68–1.09) *versus* other TKs<br>0.53 (CI 0.52–0.54)
**Triathlon**<br>Triathlon MBT uncemented	–	–	2021[a,b], 2022[a,b,c]	–	[a] HR: 1.27 (CI 1.08–1.49)<br>[b] HR: 1.38 (CI 1.16–1.65)<br>[c] HR: 1.30 (CI 1.11–1.53)
Triathlon Total Stabilizer	–	–	2022[a,b]	–	[a] HR: 2.21 (CI 1.56–3.14)<br>[b] HR: 1.80 (CI 1.16–2.79)
**Vanguard**<br>Maxim (uncemented)/Vanguard (cemented)	2020–2023	–	–	–	Revision/100 observed years:<br>1.27 (CI 0.99–1.61) *versus* other TKs<br>0.53 (CI 0.52–0.54)
Vanguard	–	–	2009[g], 2010[c], 2011[h], 2014[e], 2015[b,c,e], 2016[b,c,e], 2017[b,c,e]	–	[b] RR: 1.27 (CI 1.05–1.54)<br>[c] RR: 1.18 (CI 1.01–1.39)<br>[e] RR: 1.40 (CI 1.15–1.70)
Vanguard/Regenerex	2012, 2013	–	–	–	Revision/100 observed years: 1.73
Vanguard PS/Maxim	2014–2018	–	–	–	Revision/100 observed years: 1.04
Vanguard PS/Regenerex	2014–2023	–	–	–	Revision/100 observed years:<br>1.18 (CI 0.85–1.59) *versus* other TKs<br>0.53 (CI 0.52–0.54)
Vanguard PS/Vanguard	2019–2023	–	–	–	Revision/100 observed years:<br>0.80 (CI 0.72–0.89) *versus* other TKs<br>0.53 (CI 0.52–0.53)
Vanguard Finned	–	–	2018[a,b,c]	–	[a] RR: 1.37 (CI 1.01–1.84)<br>[b] RR: 1.48 (CI 1.03–2.14)<br>[c] RR: 1.37 (CI 1.01–1.87)

	Vanguard I–Beam		–	–	2018[b,c,e], 2019[b,e]	–		[b] RR: 1.19 (CI 1.01–1.42)<br>[e] RR: 1.29 (CI 1.09–1.54)	

[a] only for osteoarthritis;
[b] only for osteoarthritis when infection is not included;
[c] only for osteoarthritis without patella;
[d] only for osteoarthritis with patella;
[e] only for osteoarthritis without patella when infection is not included;
[f] only for osteoarthritis with patella when infection is not included;
[g] only for rheumatoid arthritis;
[h] only for rheumatoid arthritis when infection is not included.
